# Role of prostaglandin E receptor subtypes EP2 and EP4 in autocrine and paracrine functions of vascular endothelial growth factor in the inner ear

**DOI:** 10.1186/1471-2202-11-35

**Published:** 2010-03-11

**Authors:** Ryusuke Hori, Takayuki Nakagawa, Norio Yamamoto, Kiyomi Hamaguchi, Juichi Ito

**Affiliations:** 1 Department of Otolaryngology-Head and Neck Surgery, Graduate School of Medicine, Kyoto University, Kawaharacho 54, Shogoin, Sakyo-ku, 606-8507 Kyoto, Japan

## Abstract

**Background:**

The physiological effects of prostaglandin E1 (PGE1) and prostaglandin E2 (PGE2) are mediated by the prostaglandin E receptor subtypes EP1, EP2, EP3, and EP4, and the respective agonists have been purified. PGE1 and PGE2 can increase the production of vascular endothelial growth factor (VEGF), particularly through EP2 and EP4. The biological effects of VEGF are mediated by the phosphotyrosine kinase receptors fms-related tyrosine kinase-1 (Flt-1) and fetal liver kinase-1 (Flk-1). Here we examined the effects of EP2 and EP4 agonists on the production of VEGF proteins and *VEGF *messenger RNAs (mRNAs) in the inner ear, using an enzyme-linked immunosorbent assay and the real-time quantitative reverse transcription-polymerase chain reaction, respectively. We also examined the localization of EP2, VEGF, Flt-1, and Flk-1 in the cochlea by immunohistochemistry.

**Results:**

The expression of EP2 occurred in the cochlea, and the local application of an EP2 or EP4 agonist increased VEGF protein and *VEGF *mRNA levels in the inner ear. Furthermore, the intensity of the VEGF immunoreactivity in the spiral ganglion appeared to be increased by the local EP2 or EP4 agonist treatment. Immunoreactivity for Flt-1, and Flk-1 was found in the cochlear sensory epithelium, spiral ganglion, spiral ligament, and stria vascularis.

**Conclusions:**

These findings demonstrate that EP2 and EP4 agonists stimulate VEGF production in the inner ear, particularly in the spiral ganglions. Moreover, the Flt-1 and Flk-1 expression observed in the present study suggests that VEGF has autocrine and paracrine actions in the cochlea. Thus, EP2 and EP4 might be involved in the mechanisms underlying the therapeutic effects of PGE1 on acute sensorineural hearing loss via VEGF production.

## Background

Sensorineural hearing loss (SNHL) is a common disability. Once hearing has been lost, it is rarely recovered. The systemic application of corticosteroids has been accepted as the treatment of choice for acute SNHL, although its efficacy has not been substantiated [1]. In general, approximately 50% of SNHL cases show no response to the systemic application of corticosteroids [2]. Hence there is an urgent need to develop alternative treatments for acute SNHL. Prostaglandin E1 (PGE1) has often been used as one such treatment; however, its clinical efficacy remains controversial [3-5]. The physiological actions of PGE1 and prostaglandin E2 (PGE2) are mediated by the prostaglandin E receptor subtypes EP1, EP2, EP3, and EP4 [6,7]. EP2 and EP4 are coupled to G-protein stimulation, and mediate increases in cyclic adenosine monophosphate (cAMP) that activate protein kinase A (PKA) [7,8]. By contrast, the activation of EP3 decreases cAMP levels, which suggests that the selective activation of EP2 and/or EP4 might increase the clinical efficacy of PGE1 for the treatment of acute SNHL. Based on these findings, we previously examined the expression of EP4 in the cochlea, and the potential of an EP4 agonist to protect the cochlea against noise-induced trauma [9]. Our results demonstrated both EP4 expression in the cochlea, and cochlear protection against noise trauma as a result of the local application of an EP4 agonist. However, the exact mechanisms by which EP4 agonists act in the cochlea are presently unclear and require further research.

Among their various physiological and pathophysiological functions, PGE1 and PGE2 are known to increase vascular endothelial growth factor (VEGF) production via a cAMP-dependent mechanism, which is mediated by EP2 and EP4 [10-13]. VEGF, which is a 45-kDa heparin-binding homodimeric glycoprotein, plays roles in angiogenesis, vasodilation, differentiation, anti-apoptosis, proliferation, and vascular permeability in endothelial tissues [14-16]. It also has neurotrophic and neuroprotective effects in non-endothelial tissues [17-19]. Recent studies have indicated that VEGF also protects the cochlea against noise trauma [20,21]. Based on these findings, we hypothesized that VEGF is involved in the mechanisms underlying the cochlear protection against noise trauma that results from local EP4 agonist application. We thus examined the expression of EP2 in the cochlea, and the effects of local EP2 and EP4 agonist application on the modulation of VEGF in the inner ear. The current study demonstrated an increase in the levels of VEGF proteins and messenger RNAs (mRNAs) following local EP2 and EP4 agonist application according to an enzyme-linked immunosorbent assay (ELISA), immunohistochemistry, and the real-time quantitative reverse transcription-polymerase chain reaction (qRT-PCR). We also demonstrated the expression of VEGF receptor-1 (VEGFR-1) or fms-related tyrosine kinase-1 (Flt-1), and VEGF receptor-2 (VEGFR-2) or fetal liver kinase-1 (Flk-1), which mediate the physiological actions of VEGF in the cochlea [22,23].

## Results

### EP2 expression in cochleae

**Figure 1:**
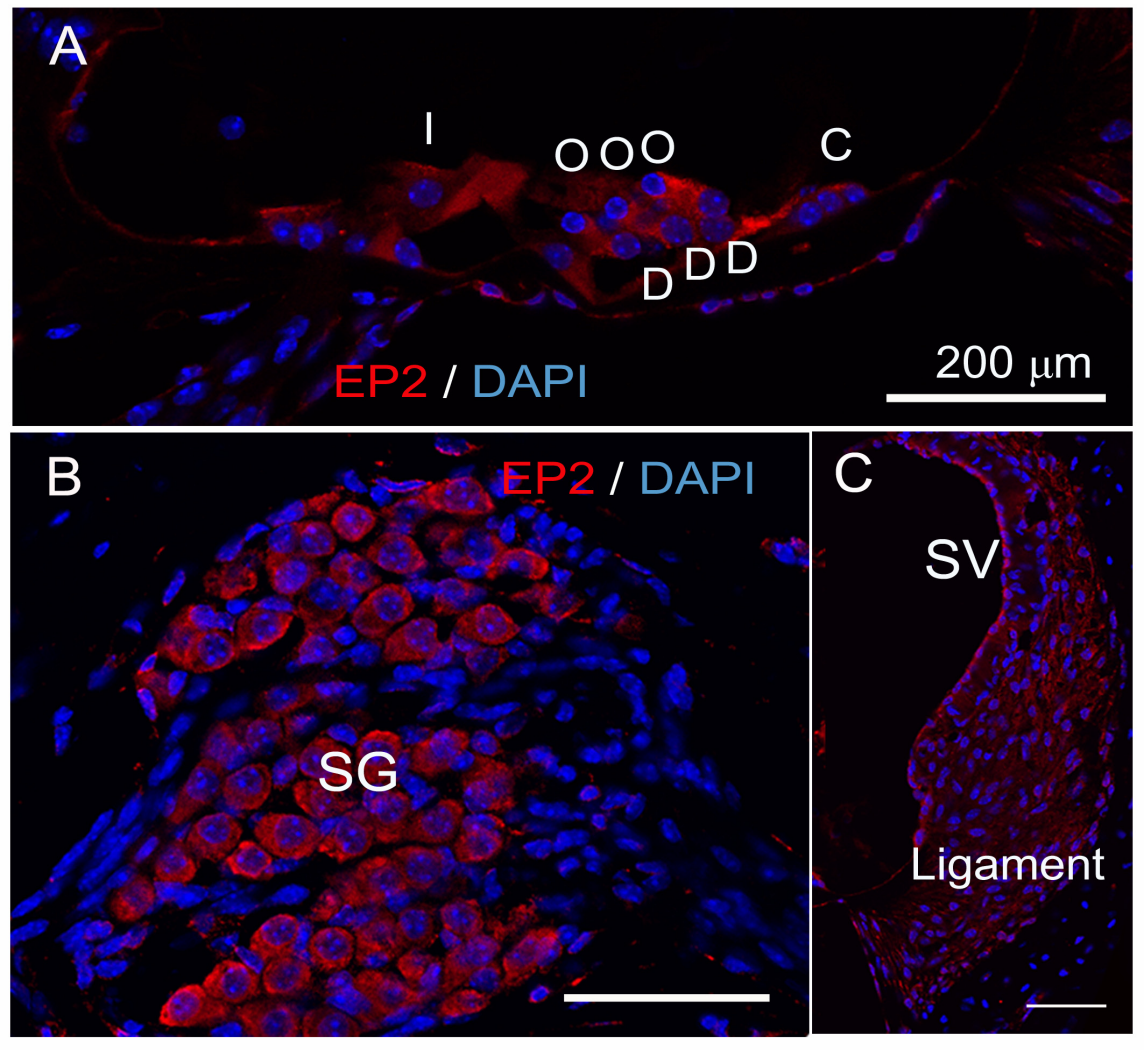
**Immunoreactivity for EP2 in the cochlea**. EP2 expression (red) was detected n the cochlear sensory epithelium (A; I, inner hair cell; O, outer hair cell; D, Deiter's cell; C, Claudius' cell), spiral ganglion neurons (B; SG), spiral ligament (C; ligament) and stria vascularis (C; SV). Nuclei were labeled with DAPI. The specimens were viewed with 63× oil objective. Scale bar = 200 μm.

Our previous study demonstrated the localization of EP4 in the mouse cochlea [9]; however, EP2 expression in the mouse cochlea has not been determined. We thus performed an immunohistochemical analysis of EP2 using normal mouse cochlear specimens in the current study. Immunostaining revealed that the EP2 expression occurred in the stria vascularis, spiral ligament, spiral ganglion neurons, supporting cells, and hair cells (Figure 1). Negative controls using a specific blocking peptide showed no immunoreactivity (data not shown). These findings confirmed that EP2 expression was present in the cochlear cells, similar to EP4 [9].

### VEGF increase due to EP2 and EP4 agonists

**Figure 2:**
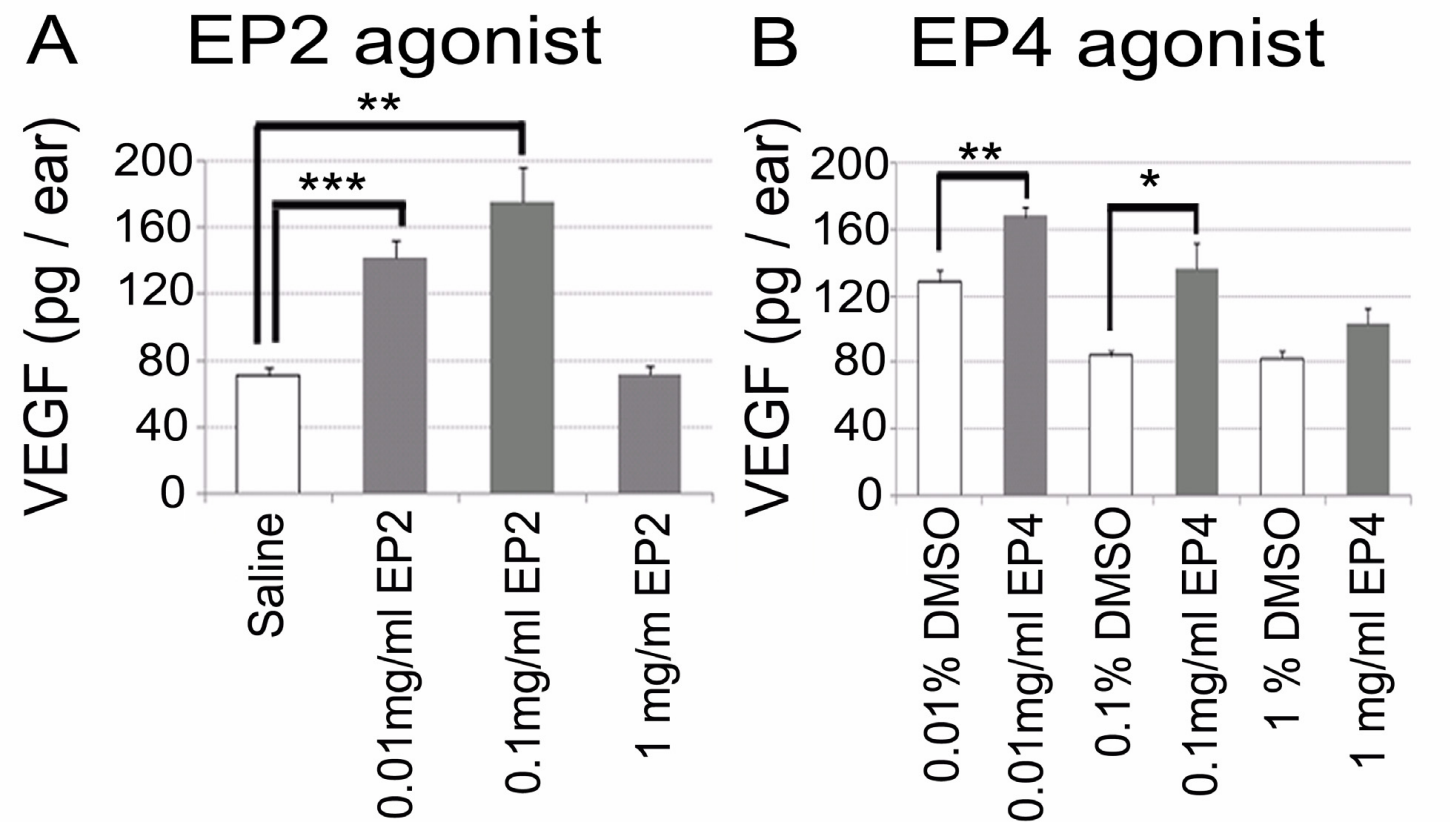
**VEGF protein levels in the inner ear measured by ELISA**. (A) VEGF protein levels in samples treated with 0.01 or 0.1 mg/ml EP2 agonist were significantly higher than those in samples treated with physiological saline (**, *p *< 0.01, and ***, *p *< 0.001, ANOVA with Scheffe's method). (B) VEGF protein levels in samples treated with 0.01 or 0.1 mg/ml EP4 agonist were significantly higher than those in samples treated with 0.01 or 0.1% DMSO, respectively (*, *p *< 0.05, **, *p *< 0.01, Student's *t*-test).

To examine the changes in the expression of VEGF proteins and *VEGF *mRNAs in the inner ear caused by EP2 and EP4 agonists, we performed ELISA and real-time qRT-PCR analyses of extracts from inner ear specimens following the local application of EP2 and EP4 agonists or control substrates. ELISA analyses revealed significant increases in VEGF protein levels following EP2 agonist application at concentrations of 0.01 and 0.1 mg/ml (Figure 2A; *p *< 0.001 and *p *= 0.005, respectively). No significant increase of VEGF protein levels was found in samples treated with 1 mg/ml EP2 agonist. The maximum VEGF protein increase was found at a concentration of 0.1 mg/ml. Local application of an EP4 agonist also caused a significant increase of VEGF protein levels in the inner ear. Specimens treated with 0.01 or 0.1 mg/ml EP4 exhibited significantly higher VEGF protein levels than those treated with 0.01 or 0.1% dimethyl sulfoxide (DMSO) (Figure 2B; *p *= 0.004 and 0.026, respectively). No significant increase of VEGF protein levels was identified in samples treated with 1 mg/ml EP4 agonist in comparison with those treated with 1% DMSO. The EP4 agonist caused a maximum VEGF protein increase at a concentration of 0.01 mg/ml.

**Figure 3:**
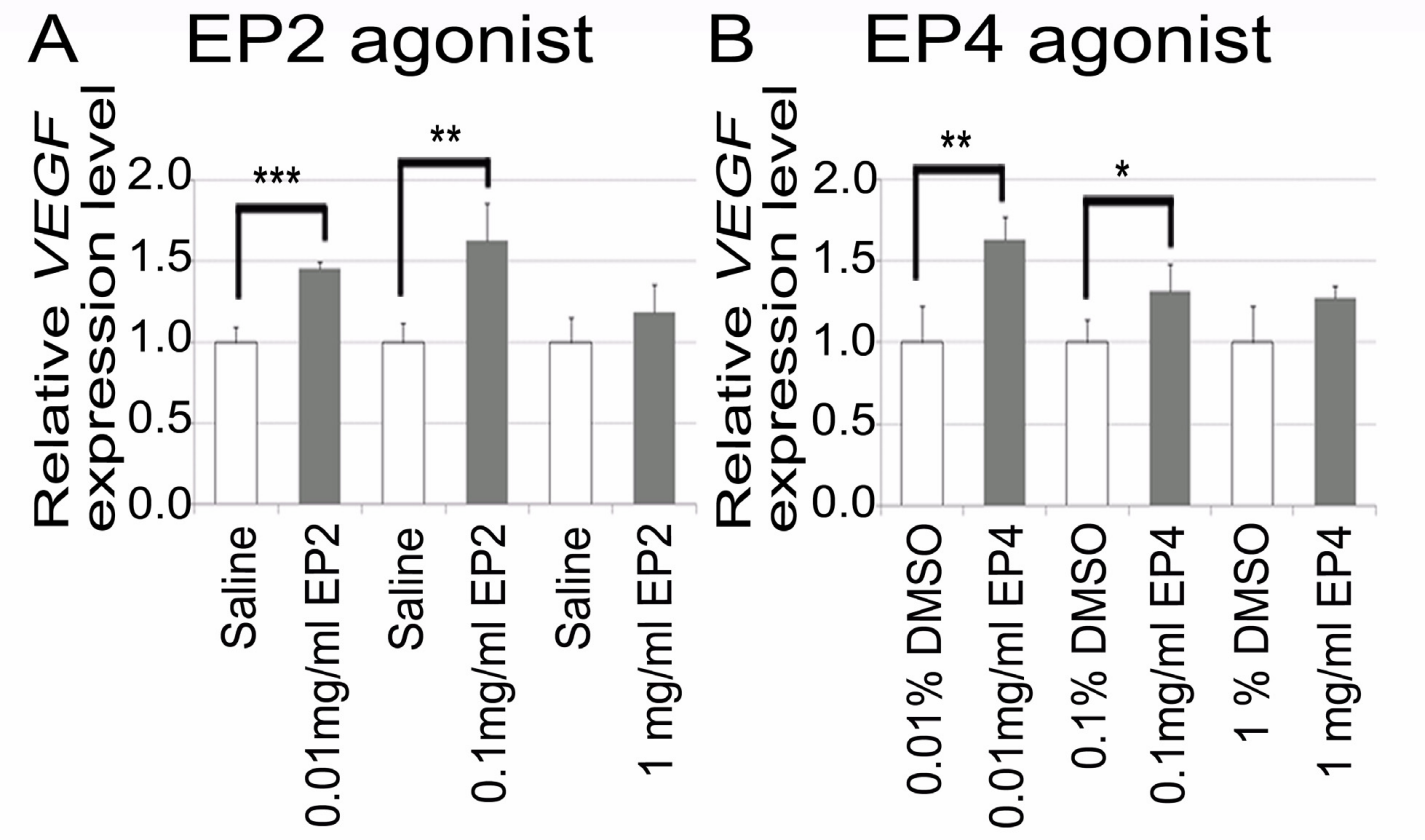
**Relative *VEGF *expression levels in inner ear according to real-time qRT-PCR**. (A) Samples treated with EP2 agonist at 0.01 or 0.1 mg/ml expressed higher levels of *VEGF *mRNAs than those treated with physiological saline. (B) Samples treated with EP4 agonist at 0.01 or 0.1 mg/ml expressed higher levels of *VEGF *mRNAs than those treated with 0.01 or 0.1% DMSO, respectively (*, *p *< 0.05, **, *p *< 0.01, and ***, *p *< 0.001, Student's *t*-test).

Real-time qRT-PCR analyses demonstrated a significant increase of *VEGF *mRNA levels following the local application of the EP2 and EP4 agonists. The relative *VEGF *expression levels in the samples treated with 0.01 or 0.1 mg/ml EP2 agonist were significantly higher (*p *< 0.001 and *p *= 0.007, respectively) than those in the control samples treated with saline (Figure 3A). There was no significant difference in the relative *VEGF *expression levels between the samples treated with 1 mg/ml EP2 agonist and the control samples, similar to the VEGF protein levels. The relative *VEGF *expression levels in the samples treated with 0.01 and 0.1 mg/ml EP4 agonist were significantly higher (*p *= 0.002 and 0.036, respectively) than those in the control samples treated with DMSO (Figure 3B). The samples treated with 1 mg/ml EP4 agonist exhibited no significant increase of *VEGF *mRNA levels in comparison with the control samples treated with DMSO, similar to the VEGF protein levels. The maximum increases in *VEGF *mRNA levels were observed after treatment with 0.1 mg/ml EP2 and 0.01 mg/ml EP4, similar to the VEGF protein levels. These results demonstrated that the EP2 and EP4 agonists stimulated VEGF production in the inner ear.

### VEGF expression in cochleae

**Figure 4:**
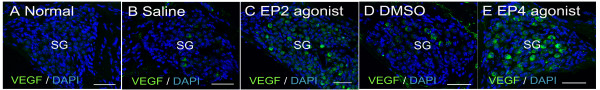
**Immunoreactivity for VEGF in the spiral ganglion cells**. Little or no staining was detected in the spiral ganglion cells of the untreated samples (A; Normal). VEGF expression (green) was weakly detected in a few spiral ganglion neurons in samples treated with physiological saline (B; Saline) and those treated with 0.1% DMSO (D; DMSO). VEGF expression was strongly detected in a large number of the spiral ganglion neurons treated with EP2 agonist (C; EP2 agonist) or EP4 agonist (E; EP4 agonist). Nuclei were labeled with DAPI. The specimens were viewed with 63× oil objective. Scale bar = 200 μm.

Our previous study [9] and immunostaining for EP2 in the present study demonstrated the presence of EP2 and EP4 in various types of cochlear cells. ELISA and real-time qRT-PCR analyses revealed VEGF induction in inner ears by local EP2 or EP4 agonist application. We then examined what type cochlear cells were responsible for VEGF increase in the cochlea in response to local application of EP2 or EP4 agonists. We thus performed immunostaining for VEGF in cochlear specimens treated with EP2 or EP4 agonist and in untreated cochlear specimens. The untreated cochlear specimens showed little or no staining for VEGF in the spiral ganglion neurons (Figure 4A), supporting cells, hair cells, spiral ligament, and stria vascularis (data not shown). In the control specimens treated with saline or DMSO, VEGF immunoreactivity was weakly detectable in some of the spiral ganglion neurons (Figure 4B, D). By contrast, intense immunoreactivity for VEGF was observed in the spiral ganglion neurons of the specimens treated with EP2 and EP4 agonists (Figure 4C, E). The other EP2-positive and EP4-positive regions of the cochlea, including the supporting cells, hair cells, spiral ligament, and stria vascularis, showed little or no immunoreactivity for VEGF under all of the experimental conditions, and no changes in VEGF immunoreactivity were observed. These findings indicate that the spiral ganglion neurons are responsible for the increase in VEGF expression in the cochlea that results from the local application of an EP2 or EP4 agonist.

### VEGFR expression in cochleae

**Figure 5:**
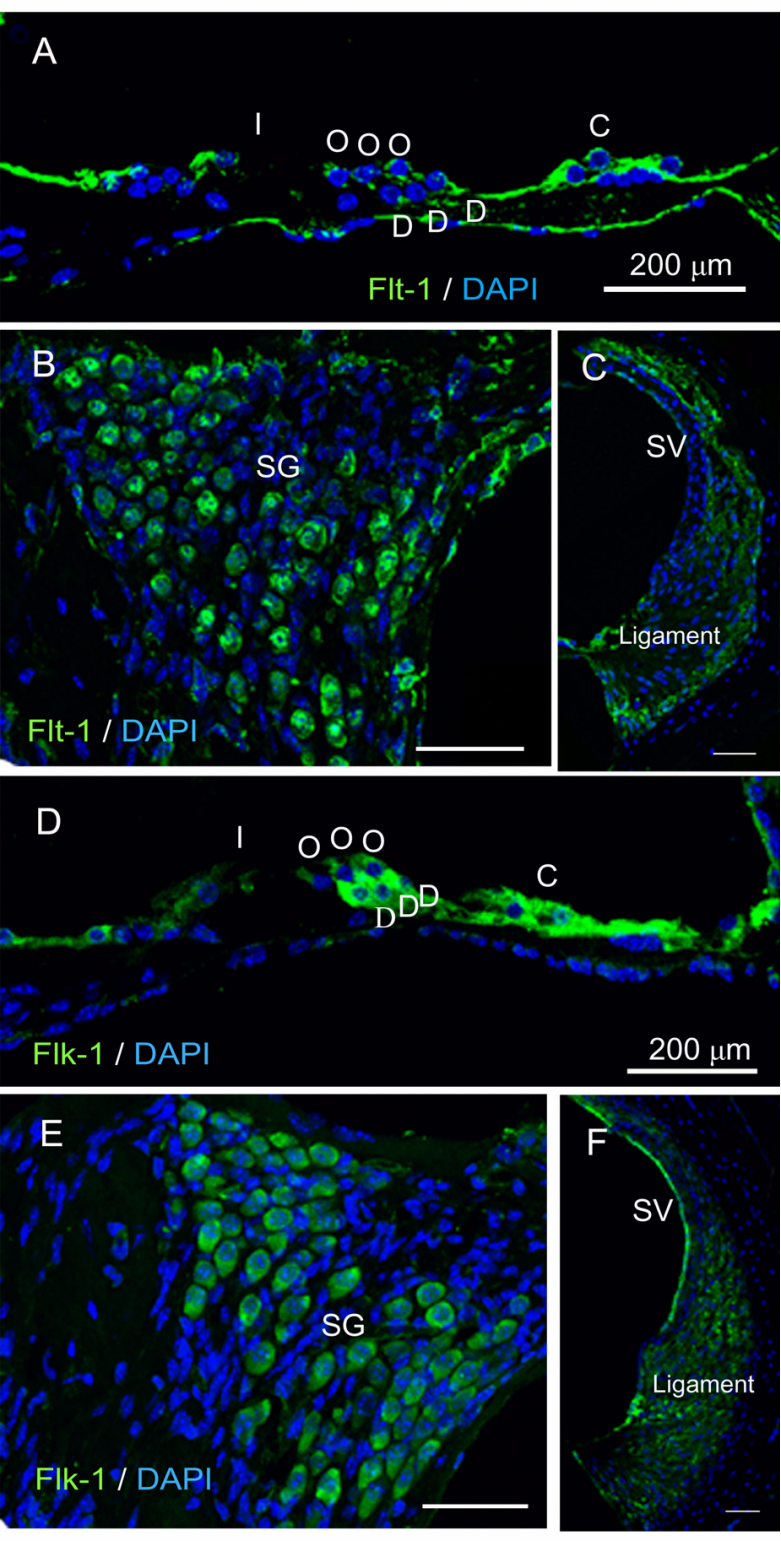
**Immunoreactivity for Flt-1 and Flk-1 in the cochlea**. (A-C) Flt-1 expression (green) was detected n the cochlear sensory epithelium (A; I, inner hair cell; O, outer hair cell; D, Deiter's cell; C, Claudius' cell), spiral ganglion neurons (B; SG) and spiral ligament (C; ligament). (D-F) Flk-1 expression was found in the cochlear sensory epithelium (D), spiral ganglion neurons (E), spiral ligament (F) and stria vascularis (F; SV). Nuclei were labeled with DAPI. The specimens were viewed with 63× oil objective. Scale bar = 200 μm.

To examine the target cells of VEGF in the cochlea, we performed immunostaining for VEGFRs, Flt-1 and Flk-1, using non-treated cochlear specimens. Flt-1 expression was detected in the supporting cells, hair cells, spiral ganglion neurons, and spiral ligament fibrocytes (Figure 5A-C). Flk-1 expression was identified in the supporting cells, hair cells, spiral ganglion neurons, spiral ligament fibrocytes, and stria vascularis marginal cells (Figure 5D-F). These findings suggest that VEGF generated in the spiral ganglion neurons in response to an EP2 or EP4 agonist acts on cells expressing Flt-1 and/or Flk-1 in the cochlea in a paracrine or autocrine fashion.

## Discussion

Recently, the prostaglandin E receptor subtypes EP1, EP2, EP3 and EP4 were cloned, and their specific agonists were purified [7]. EP2 and EP4 are coupled to G-protein stimulation and mediate increases in cAMP, while the activation of EP3 leads to a decrease of cAMP levels. We thus hypothesized that selective agonists for EP2 and EP4 might be more effective than PGE1 for the treatment of SNHL. In a previous study, we demonstrated the expression of EP4 in the cochlea, and the attenuation of noise-induced damage as a result of local EP4 agonist administration [9]. However, the mechanisms underlying the protective effect of EP4 against on cochlear damage have not previously been investigated. In the present study, we highlighted VEGF induction via EP2 or EP4 activation. The present study therefore examined the effects of EP2 and EP4 agonists on the modulation of inner ear VEGF levels.

We initially confirmed that EP2 expression occurred in the cochlea and showed a similar pattern to EP4 expression [9]. ELISA and real-time qRT-PCR analyses demonstrated an increase of VEGF protein and mRNA levels in the inner ear after local EP2 or EP4 agonist application, indicating that the stimulation of EP2 and/or EP4 activates VEGF production in the inner ear. Immunostaining for VEGF showed that the strongest response to EP2 or EP4 stimulation occurred in the spiral ganglion neurons, suggesting that they are responsible for increasing the VEGF levels in the inner ear. In addition, expression of the VEGFRs Flt-1 and Flk-1 was identified in the sensory epithelial cells, spiral ganglion neurons, spiral ligament fibrocytes, and stria vascularis marginal cells, which was consistent with previous findings [21]. These results indicate that EP2 and EP4 are involved in the mechanisms underlying the autocrine and paracrine functions of VEGF in the inner ear. For mechanisms of VEGF induction via EP2 or EP4 activation, recent studies have indicated involvement of cAMP- and PKA-dependent mechanisms via SP-1 transcription factor binding sites on the VEGF promoter [12,24,25]. However, further studies are needed to determine signaling cascades for VEGF induction via EP2 and EP4 in the inner ear.

VEGF was initially identified as a mitogen for endothelial cells, which promotes angiogenesis and vascular permeability. Numerous studies have demonstrated that VEGF plays crucial roles in the development and protection of neuronal cells [17,26]. VEGF and its receptors are also present in the cochlea, and have been shown to play significant roles in the maintenance of cochlear homeostasis [20,21,27-30]. The present findings provide new insights into the mechanisms underlying the regulation of VEGF expression in the cochlea, which is mediated by EP2 and EP4. Past work has demonstrated that hypoxia-inducible factor-1 induces VEGF upregulation in the cochlea, which is involved in the cochlear pericyte responses to noise trauma [30]. In the central nervous system, VEGF synthesized by neurons was reported to promote survival and angiogenesis, as well as autocrine and paracrine neuronal survival [31]. The present results demonstrated spiral ganglion neuron responses to EP2 and EP4 stimulation, and the expression of both Flt-1 and Flk-1 in various cochlear components including the sensory epithelium, spiral ganglion, and stria vascularis. Flk-1 appears to mediate almost all of the known cellular responses to VEGF [32], suggesting that Flk-1 also plays an essential role in VEGF function in the cochlea. However, an actual answer for this awaits further investigations. Consequently, present findings indicate that VEGF has autocrine and paracrine effects on neuronal and endothelial cells in the cochlea as well as in the central nervous system, and that EP2 and EP4 are involved in autocrine and paracrine functions of VEGF in the cochlea.

Hypoxic preconditioning is known to have protective effects on neurons [33,34]. Recently, VEGF was reported to be involved in the protective effect of neuronal preconditioning against ischemic injury [35]. Protection against noise exposure by cochlear preconditioning has also been documented [36]. VEGF upregulation in the cochlea is induced not only by intense noise exposure [20,30] but also by moderate noise exposure [21]. These findings suggest that VEGF is also involved in the mechanisms underlying protection against noise exposure by cochlear preconditioning. Our previous study demonstrated that pretreatment with an EP4 agonist led to almost complete protection of the cochleae against noise trauma [9]. In addition, our present findings demonstrate the involvement of EP2 and EP4 in the regulation of VEGF synthesis in the inner ear. EP2-mediated and EP4-mediated VEGF synthesis in the cochlea could therefore be associated with protection against noise by cochlear preconditioning. However, distinct roles of VEGF in cochlear protection by local application of EP2 or EP4 agonists have not been elucidated. Further studies are required to determine whether VEGF antagonists suppress protective effects of EP2 or EP4 agonists for cochleae.

## Conclusions

The present study demonstrated that local EP2 and EP4 agonist treatment induces VEGF synthesis in the inner ear, especially in the spiral ganglion neurons. The expression of VEGFRs was detected in the cochlear sensory epithelium, spiral ganglion, spiral ligament, and stria vascularis. These findings indicate the involvement of EP2 and EP4 in the autocrine and paracrine functions of VEGF in the cochlea.

## Methods

### Animals

Male C57BL/6 mice at 8 weeks of age were purchased from Japan SLC, Inc. (Hamamatsu, Japan). The Animal Research Committee of the Graduate School of Medicine, Kyoto University, Japan, approved all of the experimental protocols. Animal care was supervised by the Institute of Laboratory Animals of the Graduate School of Medicine, Kyoto University. All of the experimental procedures were performed in accordance with the National Institutes of Health (NIH) Guide for the Care and Use of Laboratory Animals.

### EP2 expression in cochleae

The expression of EP2 in the cochlea was immunohistochemically examined using normal cochlear specimens (n = 3). Under general anesthesia with midazolam (10 mg/kg; Astellas, Tokyo, Japan) and xylazine (10 mg/kg; Bayer, Tokyo, Japan), the animals were transcardially perfused with 0.01 M phosphate-buffered saline (PBS; pH 7.4) followed by 4% paraformaldehyde (PFA) in PBS. The temporal bones were immediately dissected out and immersed in the same fixative for 2 h at 4°C. After decalcification with 0.1 M ethylenediamine tetra-acetic acid (EDTA) for 7 days at 4°C, 10-μm-thick cryostat sections were prepared. Two midmodiolus sections from each cochlea were subjected to immunostaining for EP2. Anti-EP2 polyclonal antibody (dilution, 1:250; Cayman Chemical, Ann Arbor, MI, USA) was used as the primary antibody, and Alexa 568-conjugated goat anti-rabbit immunoglobulin G (IgG; dilution, 1:500; Invitrogen, Carlsbad, CA, USA) was used as the secondary antibody. After immunostaining for EP2, the nuclei were counterstained with 4,6-diamidino,2-phenylindole dihydrochloride (DAPI; 1 μg/ml in PBS; Molecular Probes, Eugene, OR, USA). Heart specimens obtained from the mice were used as positive controls for EP2. Nonspecific labeling was tested by blocking protein-antibody complex formation using EP2-blocking peptide (Cayman Chemical). The specimens were viewed with a Leica TCS-SPE confocal microscope (Leica Microsystems, Wetzlar, Germany).

### Local application of EP2 or EP4 agonist to inner ears

The EP2 agonist ONO-AE1-259-01 and the EP4 agonist ONO-AE1-329 (both from Ono Pharmaceutical Co., Ltd., Osaka, Japan) were used in this study. The EP2 agonist was dissolved in physiological saline to give a final concentration of 0.01, 0.1, or 1 mg/ml. The EP4 agonist was dissolved in DMSO and diluted with physiological saline to give a final concentration of 0.01, 0.1, or 1 mg/ml containing 0.01, 0.1, or 1% DMSO, respectively. Controls for EP2 agonist application were treated with saline, and controls for EP4 agonist application were treated with 0.01, 0.1, or 1% DMSO. Drug application was performed under general anesthesia with midazolam and xylazine as previously reported [37-39]. A retroauricular incision was made in the left ear, and the posterior semicircular canal (PSCC) was exposed. A small hole was made in the bony wall of the PSCC. A fused silica glass needle (EiCOM, Kyoto, Japan) was then inserted into the perilymphatic space of the PSCC, and the appropriate material was injected at a rate of 0.5 μl/min for 4 min (total = 2 μl) using a micro syringe pump (EiCOM). At 24 h after the injection into the PSCC, the animals were transcardially perfused with PBS under general anesthesia with midazolam and xylazine, and the inner ears were immediately dissected out. The inner ear samples were subjected to ELISA or real-time qRT-PCR analysis.

### Quantitative assessments of VEGF

ELISA and real-time qRT-PCR analyses were employed to assess the levels of VEGF proteins and mRNAs in the inner ears treated with 0.01, 0.1, or 1 mg/ml EP2 agonist, physiological saline, 0.01, 0.1, or 1 mg/ml EP4 agonist, or 0.01, 0.1, or 1% DMSO (n = 5 for each condition). ELISA analyses for VEGF proteins were performed using a mouse VEGF assay kit (Immuno-Biological Laboratories Co., Ltd., Takasaki, Japan) according to the manufacturer's protocol. All reactions were performed in triplicate.

For the real-time qRT-PCR analyses of *VEGF *mRNAs, the inner ear samples were homogenized with Trizol (Invitrogen), and total RNA was extracted using an RNeasy mini kit (Qiagen Ltd., Valencia, CA, USA). To eliminate genomic DNA contamination, the total RNA was treated with DNaseI (Ambion, Austin, TX, USA). The quantity and quality were evaluated using the A260/280 ratio and the appearance of the 18S and 28S ribosomal RNA bands upon electrophoresis. Complementary DNA (cDNA) was synthesized by reverse transcription using TaqMan RT reagents (Applied Biosystems Inc., Foster City, CA, USA). The reactions were performed using a GeneAmp PCR system 9700 (Applied Biosystems Inc.) under the following conditions: 25°C for 10 min, 48°C for 30 min, 95°C for 5 min, and 4°C for 5 min. The primers used in the real-time qRT-PCR for *VEGF *and for the housekeeping gene glyceraldehydes 3-phosphate dehydrogenase (*GAPDH*) were as follows: 5'-ACTTGTGTTGGGAGGAGGA-3' (sense) and 5'-AAAGGACTTCGGCCTCTCT-3' (antisense) for *VEGF *(97 base pairs [bp]); and 5'-TGTGTCCGTCGTGGATCTGA-3' (sense) and 5'-CCTGCTTCACCACCTTCTTGAT-3' (antisense) for *GAPDH *(77 bp). The real-time qRT-PCR was performed at a final volume of 20 μl according to the manufacturer's protocols. The reaction mix comprised template cDNA, 10 μl POWER SYBR Green Master Mix (Applied Biosystems Inc.), a 0.5-μM final concentration of each primer, and ribonuclease-free water. The amplification conditions were as follows: 50°C for 2 min, 95°C for 10 min, 40 cycles of denaturation at 95°C for 15 s, and annealing at 60°C for 1 min. Fluorescence was detected with the ABI Prism 7000 sequence detection system (Applied Biosystems Inc.) and the associated software. Negative controls without template were included, and all reactions were performed in triplicate. The *VEGF *expression values were normalized to the housekeeping gene by dividing the mean of the *VEGF *triplicate value by the mean of the *GAPDH *triplicate value.

### VEGF expression in cochleae

Immunohistochemistry was performed to examine the localization of VEGF in cochlear specimens treated with 0.1 mg/ml EP2 agonist, physiological saline, 0.1 mg/ml EP4 agonist, or 0.1% DMSO (n = 4 in each condition), and in non-treated cochleae (n = 3). Two mid modiolus sections from each cochlea were subjected to immunostaining for VEGF. Anti-mouse VEGF antibody (dilution, 5 μg/ml; R&D systems Inc., Minneapolis, MN, USA) was used as the primary antibody, and Alexa 488-conjugated donkey anti-goat IgG (dilution, 1:500; Invitrogen) was used as the secondary antibody. Kidney specimens obtained from the mice were used as positive controls. Nonspecific labeling was tested by omitting the primary antibody from the staining procedures.

### VEGFR expression in cochleae

The expression of the VEGFRs Flt-1 and Flk-1 in normal cochleae (n = 4) was examined by immunohistochemistry. Anti-mouse Flt-1 antibody (dilution, 25 μg/ml; R&D Systems Inc.) and anti-mouse Flk-1 antibody (dilution, 15 μg/ml; R&D systems Inc.) were used as the primary antibodies, and Alexa 488-conjugated goat anti-rat IgG (dilution, 1:500; Invitrogen) and Alexa 488-conjugated donkey anti-goat IgG (dilution, 1:500; Invitrogen) were used as the secondary antibodies for Flt-1 and for Flk-1, respectively. The positive and negative controls were the same as those used in the VEGF immunostaining.

### Statistical analysis

The differences in the levels of VEGF proteins among the samples treated with EP2 were examined using the one-factor analysis of variance with Scheffe's method. The differences in the levels of VEGF proteins between the samples treated with EP4 agonist and the control samples and those in the levels of and *VEGF *mRNAs between between the samples treated with EP2 or EP4 agonist and the control samples were examined using the unpaired *t*-test. A *p *value < 0.05 was considered statistically significant. All data are represented as the mean ± standard error.

## Abbreviations

bp: base pairs; cAMP: cyclic adenosine monophosphate; cDNA: complementary DNA; DAPI: 4,6-diamidino, 2-phenylindole dihydrochloride; DMSO: dimethyl sulfoxide; EDTA: ethylenediamine tetra-acetic acid; ELISA: enzyme-linked immunosorbent assay; EP: prostaglandin E receptor subtype; Flk-1: fetal liver kinase-1; Flt-1: fms-related tyrosine kinase-1; *GAPDH*: glyceraldehyde 3-phosphate dehydrogenase; IgG: immunoglobulin G; mRNA: messenger RNA; PBS: phosphate-buffered saline; PFA: paraformaldehyde; PGE1: prostaglandin E1; PGE2: prostaglandin E2; PKA: protein kinase A; PSCC: posterior semicircular canal; qRT-PCR: quantitative reverse transcription-polymerase chain reaction; SNHL: sensorineural hearing loss; VEGF: vascular endothelial growth factor; VEGFR-1: vascular endothelial growth factor receptor-1; VEGFR-2: vascular endothelial growth factor receptor-2.

## Authors' contributions

RH performed the experiments and data analyses, and contributed to manuscript preparation. TN coordinated and guided the experimental plans and contributed to manuscript writing. KH co-worked on the local application of EP agonists to the inner ear. NY and JI contributed to the conception of this study and participated in its design and execution. All authors contributed to and approved the final manuscript.
